# Sequence-based analysis of the genus *Ruminococcus* resolves its phylogeny and reveals strong host association

**DOI:** 10.1099/mgen.0.000099

**Published:** 2016-12-12

**Authors:** Alex J. La Reau, Jan P. Meier-Kolthoff, Garret Suen

**Affiliations:** ^1^​ Department of Bacteriology, University of Wisconsin – Madison, Madison, WI 53706, USA; ^2^​ Leibniz Institute DSMZ – German Collection of Microorganisms and Cell Cultures, 38124 Braunschweig, Germany

**Keywords:** *Ruminococcus*, host range, phylogeny, phylogenomics, 16S rRNA, host diet

## Abstract

It has become increasingly clear that the composition of mammalian gut microbial communities is substantially diet driven. These microbiota form intricate mutualisms with their hosts, which have profound implications on overall health. For example, many gut microbes are involved in the conversion of host-ingested dietary polysaccharides into host-usable nutrients. One group of important gut microbial symbionts are bacteria in the genus *Ruminococcus*. Originally isolated from the bovine rumen, ruminococci have been found in numerous mammalian hosts, including other ruminants, and non-ruminants such as horses, pigs and humans. All ruminococci require fermentable carbohydrates for growth, and their substrate preferences appear to be based on the diet of their particular host. Most ruminococci that have been studied are those capable of degrading cellulose, much less is known about non-cellulolytic non-ruminant-associated species, and even less is known about the environmental distribution of ruminococci as a whole. Here, we capitalized on the wealth of publicly available 16S rRNA gene sequences, genomes and large-scale microbiota studies to both resolve the phylogenetic placement of described species in the genus *Ruminococcus*, and further demonstrate that this genus has largely unexplored diversity and a staggering host distribution. We present evidence that ruminococci are predominantly associated with herbivores and omnivores, and our data supports the hypothesis that very few ruminococci are found consistently in non-host-associated environments. This study not only helps to resolve the phylogeny of this important genus, but also provides a framework for understanding its distribution in natural systems.

## Data Summary

The 16S rRNA and *recA* gene sequences used for the single and dual-locus phylogenetic analyses were obtained from the National Center for Biotechnology Information and are detailed in Table S1(available in the online Supplementary Material). The single-locus phylogenetic tree of the *recA* gene can be found in Figure S1 (available in the online Supplementary Material). The 56 genomes of *Ruminococcus* and related genera used for the phylogenomic analysis were obtained from the US Department of Energy Joint Genome Institute’s Integrated Microbial Genomes and Microbiomes database, and are detailed in Table S2, and the core set of genes from these genomes is detailed in Table S3 (available in the online Supplementary Material). An additional genome was added to this set (*Eubacterium contortum* ATCC 25540) for the genome blast distance phylogeny analysis and was obtained from the PATRIC database (genome ID 39482.3; see Data Bibliography). All pairwise digital DNA-DNA hybridization values between genomes used in the phylogenomic analysis are detailed in Table S4 (available in the online Supplementary Material). A genome blast distance phylogeny of the nucleotide sequences of these genomes can be found in Figure S2 (available in the online Supplementary Material). The 16S rRNA gene sequences used in the phylogenetic analysis of undescribed ruminococci are detailed in Table S5 (available in the online Supplementary Material). The 47 microbiota studies used for the *Ruminococcus* distributional analyses are detailed in Table S6 (available in the online Supplementary Material; also see Data Bibliography), while the raw proportional data by host is detailed in Table S7. All pairwise comparisons of percent relatedness for Ruminococcus spp. and select related species are detailed in Table S8 (available in the online Supplementary Material).

## Impact Statement

In this study, we used the wealth of publicly available 16S rRNA, genomic and large-scale microbiota sequence data to address fundamental questions about the diversity, phylogenetic relationships and environmental distribution of the genus *Ruminococcus*. Ruminococci have been studied for decades since their discovery, but very little work has been done to resolve the phylogeny of these bacteria. Furthermore, all described isolates have been obtained from host sources, leading to the hypothesis that the genus is strictly host associated. To this end, we leveraged public sequence databases to generate multiple phylogenies of currently described *Ruminococcus* spp. and resolved the evolutionary relatedness of species members, in addition to identifying clades containing potentially novel isolates. We also used numerous microbiota studies to explore the environmental distribution of *Ruminococcus* sequences to show that this genus is highly diverse, and that novel species likely exist in disparate host environments. Finally, we provide strong evidence that *Ruminococcus* is a strictly host-associated genus, due to its virtual absence in all environmental datasets considered.

## Introduction

Recent work has demonstrated that the composition of the gut microbiota of mammals is substantially diet driven, with herbivores, omnivores and carnivores harbouring distinct microbial communities (Ley *et al.*, 2008a). These communities are often dominated by bacteria in the phyla *Firmicutes* and *Bacteroidetes*, which are known to form intricate mutualisms with their hosts. Importantly, these bacteria have profound implications for host health (Ley *et al.*, 2008b), such as in humans where they modulate metabolism (Li *et al.*, 2008) and immune system function (Round & Mazmanian, 2009). In other systems like herbivorous ruminants, these microbial communities degrade and ferment dietary cellulosic-based biomass into nutritive short-chain fatty acids (Flint *et al.*, 2008). One important member of both ruminant and human microbial communities is the bacterial genus *Ruminococcus*. For example, the abundance of *Ruminococcus bromii* in humans has been shown to be stimulated by a diet high in resistant starch (Walker *et al.*, 2011). Moreover, some members are now considered as ‘keystone’ species (Ze *et al.*, 2012; Moraïs *et al.*, 2016), and several occur as prominent members of the ‘core gut microbiome’ found in a majority of humans (Qin *et al.*, 2010). Aside from their presence in humans, other members are abundant and active in the degradation and fermentation of dietary polysaccharides in ruminant mammals (Leschine, 1995).

*Ruminococcus* species are defined as strictly anaerobic, Gram-positive, non-motile cocci that do not produce endospores and require fermentable carbohydrates for growth (Rainey, 2009b). They were initially described from the isolation of *Ruminococcus flavefaciens* from the bovine rumen (Sijpesteijn, 1948). *Ruminococcus* is currently considered a polyphyletic genus, with species members belonging to two separate families: the *Ruminococcaceae* and the *Lachnospiraceae* (Rainey & Janssen, 1995). The type species of the genus, *R. flavefaciens*, belongs to the *Ruminococcaceae*. Moreover, several former *Ruminococcus* species have been reclassified to the genus *Blautia* (family *Lachnospiraceae*), based on 16S rRNA gene sequence data (Lawson & Finegold, 2015; Liu *et al.*, 2008), and it has been suggested that only species of the *Ruminococcaceae* be considered as ‘true *ruminococci*’ (Rainey & Janssen, 1995).

Under this definition, there are only six described species of *Ruminococcus* to date. Some species are cellulolytic, including the rumen isolates *R. flavefaciens* and *Ruminococcus* *albus* (Hungate, 1957), and the recently described human isolate *Ruminococcus* *champanellensis* (Chassard *et al.*, 2012), which is the only known bacterial species isolated from the human colon capable of degrading crystalline cellulose (Moraïs *et al.*, 2016). Others are non-cellulolytic and utilize polysaccharides like resistant starches in the case of *R. bromii* (Ze *et al.*, 2012), or selectively use various plant hemicelluloses in the case of *Ruminococcus* *callidus* (Lay *et al.*, 2005) and ‘*Ruminococcus* *bicirculans*’ (Wegmann *et al.*, 2014). Substantial work has been done on cellulolytic *Ruminococcus* isolates from the bovine rumen due to their potential application in biofuels and their importance in animal health (Dassa *et al.*, 2014; Christopherson *et al.*, 2014; Pavlostathis *et al.*, 1988). Strains from other ruminant (Orpin *et al.*, 1985; Krause *et al.*, 1999) and non-ruminant (Julliand *et al.*, 1999) sources have also been described, but no isolate has been reported from a non-host (i.e. environmental) source to date. Presently, much less is known about the non-cellulolytic, non-ruminant, host-associated ruminococci isolates.

Here, we used publicly available 16S rRNA sequences, genomes and microbiota data to demonstrate that the genus *Ruminococcus* has unexplored diversity and a broad host distribution. Our phylogenomic analysis of the genomes of *Ruminococcus* spp. and several related taxa confirms the polyphyletic nature of the genus, with ruminococci falling into distinct, distantly related clades (i.e. the *Ruminococcaceae* and *Lachnospiraceae*). We also present evidence that *Ruminococcus* species are predominantly associated with herbivores and omnivores, relative to carnivores, and that significantly abundant *Ruminococcus* populations are absent in non-host-associated environments.

## Methods

### Dual-locus phylogenetic analysis of described *Ruminococcus* spp.

Full-length or near full-length sequences of two highly conserved genes – 16S rRNA and *recA* – were obtained for each formally described *Ruminococcus* type species (both *Ruminococcaceae* and *Lachnospiraceae*), other related genera (see Table S1 for details) and the outgroup species *Eubacterium acidaminophilum* from GenBank. In the case that both a 16S rRNA and *recA* gene sequence were not available for a type strain, another strain of the same species was used. Gene sequences for *recA* were obtained from genome data for each strain when they were not available as stand-alone sequences in GenBank.

Full-length and near full-length sequences were grouped into three libraries: 16S rRNA, *recA*, and a combined 16S rRNA and *recA* library. The single-locus libraries were imported into mega6 (Tamura *et al.*, 2013), aligned using clustalw with default parameters and trimmed to 1282 and 959 bp for the 16S rRNA and *recA* genes, respectively. A dual-locus library was created by concatenating the two alignments into a 16S rRNA–*recA* alignment. All alignments were then exported into MrBayes (v3.2.3) (Huelsenbeck & Ronquist, 2001; Ronquist & Huelsenbeck, 2003) and Bayesian phylogenetic analyses were performed (ngen=10 000 000) on each library. The resulting trees were visualized using FigTree v1.4.2 (A. Rambaut; http://tree.bio.ed.ac.uk/software/figtree).

### Phylogenomic analyses of ruminococci and related genera.

The available genome sequences for bacteria characterized as ruminococci, as well as for several related non-ruminococci (Table S2), were analysed using the US Department of Energy Joint Genome Institute’s Integrated Microbial Genomes and Microbiomes (IMG/M) database (Markowitz *et al.*, 2012). All predicted genes in the genome sequence for *R. albus* 7 (Suen *et al.*, 2011) (IMG/M Genome ID: 649633094) were used as a reference to conduct an analysis using the Phylogenetic Profiler for Single Genes tool against all other genomes, using default settings, to generate a list of orthologues shared between these genomes (Table S3). All nucleotide sequences for orthologues shared between each genome were obtained from the IMG/M database, concatenated and aligned using mafft version 7 (Katoh *et al.*, 2002; Katoh & Standley, 2013). A Bayesian phylogeny was then generated from this alignment using MrBayes (ngen=100 000).

A whole-genome-sequence-based phylogenomic analysis was conducted for those genomes obtained from the IMG/M database at the nucleotide level using the genome blast distance phylogeny (GBDP) method (Henz *et al.*, 2005; Meier-Kolthoff *et al.*, 2013a), including the inference of branch support (Meier-Kolthoff *et al.*, 2014). blast+ (Camacho *et al.*, 2009) was used as local alignment tool with default settings, and subsequent calculations of intergenomic distances were carried out with an *e*-value filter of 10^−8^, the trimming algorithm and formula *d*_5_ (Meier-Kolthoff *et al.*, 2014). The same settings were used for a complementary GBDP analysis of the entire sets of genes at the amino acid level, as conducted in an earlier study (Lagkouvardos *et al.*, 2016). ORF calling was carried out using the gene finding program Prodigal (Hyatt *et al.*, 2010). All balanced minimum evolution (BME) trees were reconstructed via fastme 2.1.4 with spr postprocessing (Lefort *et al.*, 2015). To further infer potential affiliation to the same species, all pairwise digital DNA–DNA hybridization (dDDH) values and their confidence intervals were calculated with the Genome-to-Genome Distance Calculator (ggdc 2.1; freely available at http://ggdc.dsmz.de) (Meier-Kolthoff *et al.*, 2013a) under the National Center for Biotechnology Information (NCBI)-blast setting (Table S4).

### Phylogenetic analysis of undescribed ruminococci.

A search was performed through the NCBI nucleotide sequence database using the search terms, ‘*Ruminococcus* [ORGANISM] AND 16S [TEXT WORD] AND 250:1600 [SEQUENCE LENGTH]’ (on 2/3/2015) to obtain all 16S rRNA gene sequences classified to the genus. Sequences representing contigs from whole-genome shotgun projects were removed to reduce redundancy, leaving a final *Ruminococcus* sequence library of 345 sequences. The closelyrelated *Eubacterium acidaminophilum* was used as an outgroup. The full sequence dataset is presented in Table S5.

This library was then processed in mothur (v.1.35.1) (Schloss *et al.*, 2009) using the following commands (indicated in italics) with default parameters except where indicated. Sequences were aligned (*align.seqs, flip=t*) to the Silva 16S/18S rRNA non-redundant sequence database (SSU ref NR; release 119; 534 968 total sequences). Sequences ≥900 bp in length (*screen.seqs*) were retained, followed by removal of duplicate sequences (*unique.seqs*), leaving 160 total sequences. Aligned sequences were then filtered (*filter.seqs*) and trimmed to 807 bp in length. A distance matrix was created (*dist.seqs*), and used to estimate the number of operational taxonomic units (OTUs) at various percentage-similarity cut-offs (*cluster, method=furthest*). A representative sequence for each OTU was chosen (*get.oturep*) and used to construct a Bayesian tree of OTUs at 97 % similarity in MrBayes (ngen=10 000 000).

### Mining of published datasets for *Ruminococcus* 16S rRNA gene sequences.

A broad survey of 16S rRNA microbiota sequencing studies encompassing host-associated (various animal and plant hosts) and non-host-associated (marine, freshwater and soil) environments was performed. These studies varied in sequencing methodology (i.e. 16S rRNA clone libraries, 454 pyrosequencing and Illumina platforms), and are detailed in Table S6. Raw sequences were obtained for each study and imported into separate sequence libraries. Each library was then processed in mothur by first aligning the sequences as described above, followed by a taxonomic classification (*classify.seqs*) using default parameters and the Silva reference taxonomy provided through mothur. A classification to the genus *Ruminococcus* (of family *Ruminococcaceae*) for a sequence was considered positive only for bootstrap values ≥80 (Wang *et al.*, 2007). Since the studies varied in sequencing methodologies, we could not compare relative abundances across sequence sources, but rather we determined the overall trends of distribution using proportional data for the presence of *Ruminococcus* sequences for each dataset (total *Ruminococcus* sequences/total sequences in dataset). These data are detailed in Table S7.

## Results

### Resolved phylogeny of the ruminococci

**Fig. 1.**
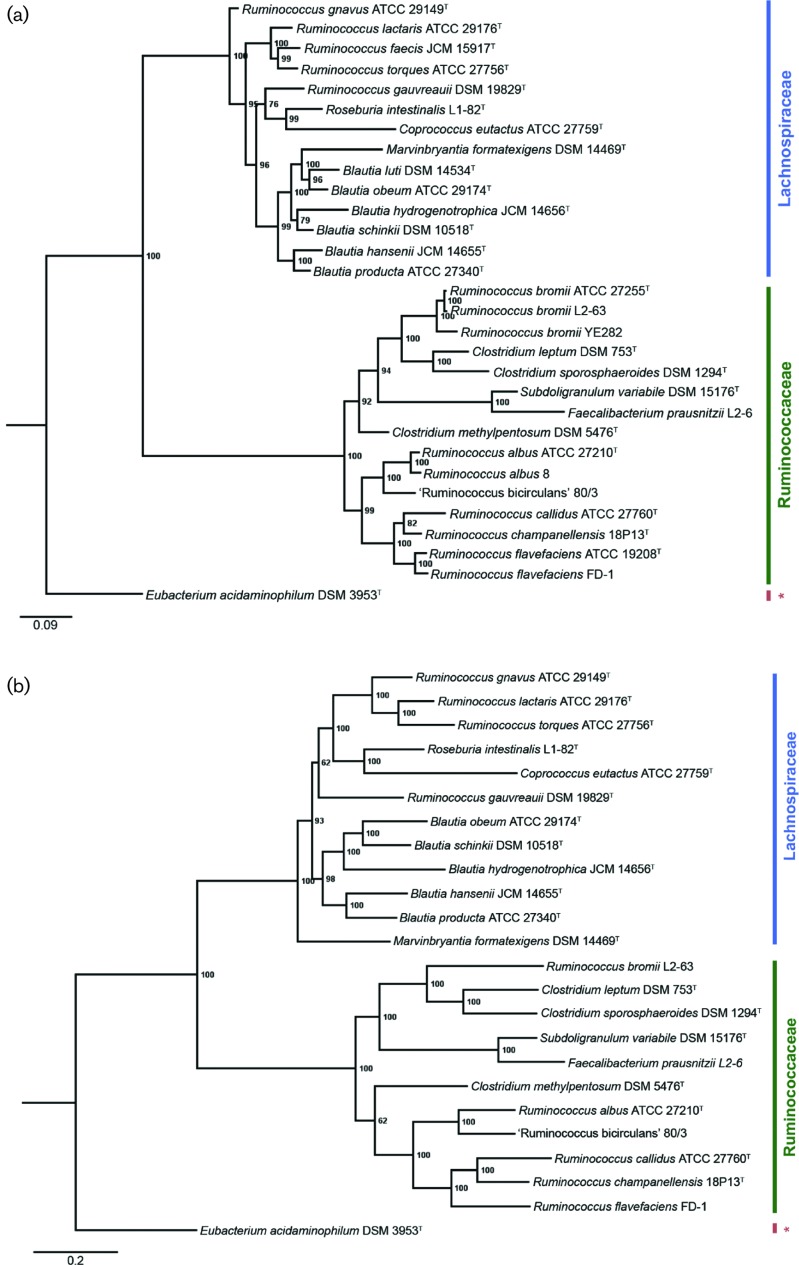

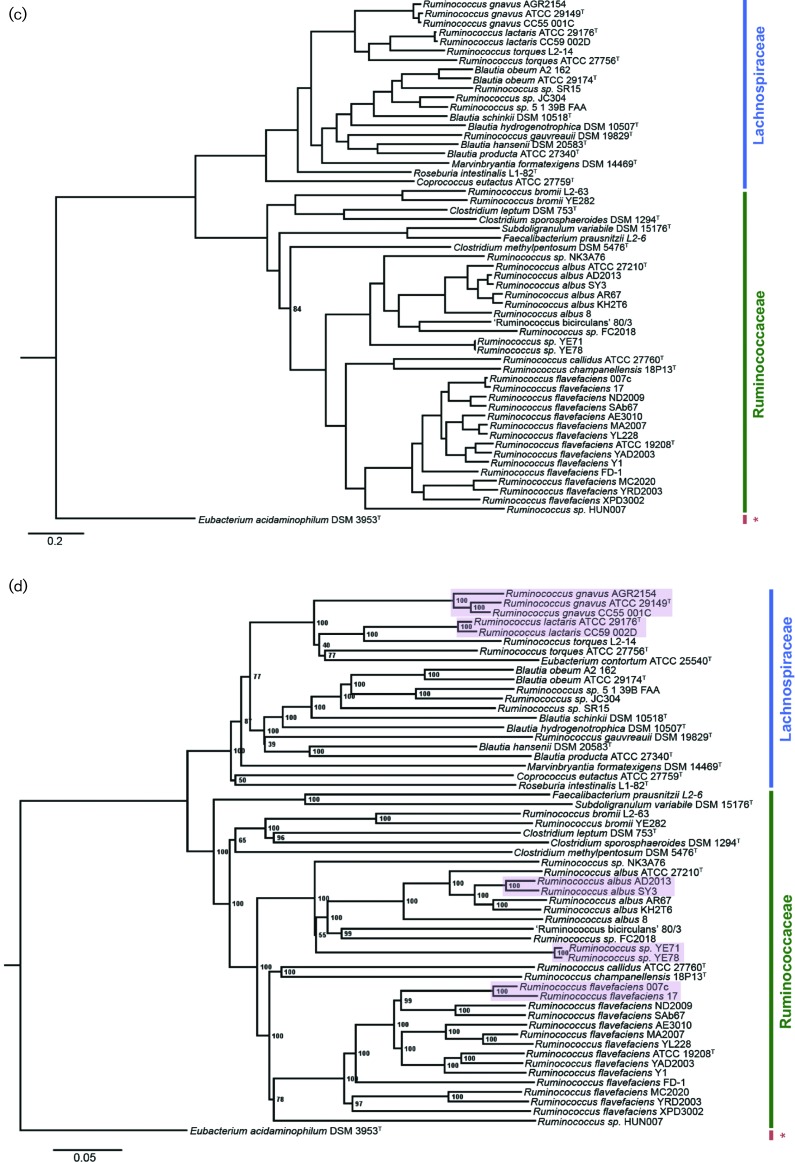
(a) A rooted Bayesian phylogeny (ngen=10 000 000) of 16S rRNA gene sequences of *Ruminococcus* spp. and related genera. (b) A rooted Bayesian phylogeny (ngen=10 000 000) of concatenated 16S rRNA and *recA* gene sequences for *Ruminococcus* spp. and related genera. (c) A rooted multi-locus Bayesian phylogeny (ngen=100 000) of 275 concatenated orthologous genes (see Table S7) shared among 56 genomes of *Ruminococcus* and closely related species available in the Integrated Microbial Genomes database. (d) A BME tree inferred by phylogenomic GBDP analysis at the amino acid level for coding genes in genomes of *Ruminococcus* spp. and related genera. All posterior probability values <100 are shown on Bayesian phylogenies a–c, and all branch support values are shown for the GBDP analysis (mean support of 93.7 % for the full tree). All type strains are indicated with a superscript T. The red asterisks indicates family *Eubacteriaceae* for *E. acidaminophilum*, the outgroup in all trees presented. The purple highlighting indicates taxa inferred as the same species according to the GBDP analysis.

In order to obtain a highly resolved view of the relationship of all currently described ‘*Ruminococcus’* species, we created four separate phylogenetic trees at varying levels of genomic resolution (Fig. 1) including: (a) a Bayesian phylogenetic tree of 16S rRNA gene sequences, (b) a dual-locus Bayesian phylogenetic tree using the 16S rRNA and *recA* gene sequences, (c) a phylogenomic Bayesian tree using 275 orthologues shared amongst 41 *Ruminococcus* genomes and 15 genomes of taxa from closely related genera, and (d) a BME GBDP analysis of the entire set of genes at the amino acid level for all genomes. All four trees showed confirmation of the reported split between *Ruminococcaceae* (Rainey, 2009b) and *Lachnospiraceae* (Rainey, 2009a; Cornick & Stanton, 2009) species in the genus *Ruminococcus* (Rainey & Janssen, 1995) with high confidence (i.e. high posterior probability values at each node for Bayesian trees and high branch support values for the BME tree with a mean of 93.7 %). These findings were also separately confirmed for the *recA* single-locus tree (Fig. S1). Moreover, these trees detailed the phylogenetic relationship between all described *Ruminococcus* species within both families. For example, the true ruminococci all form a monophyletic group within the *Ruminococcaceae* in all of our trees with the exception of *R. bromii* strains, which are deeply rooted and form a clade with more closely related *Clostridium* spp. Furthermore, the ruminococci that were previously reclassified to the genus *Blautia* all form a monophyletic clade with each other, *Ruminococcus gauvreauii* and several other undescribed *Ruminococcus* spp. (Fig. 1b–d), although this is not as resolved at the 16S rRNA level alone (Fig. 1a). All trees showed nearly identical topologies for all taxa examined. However, minor topological changes were seen for the GBDP nucleotide analysis (Fig. S2) in which some *Ruminococcaceae* taxa were found to group in the *Lachnospiraceae* clade, though branch support values for these discrepancies were low.

Our phylogenomic analysis revealed that the 275 orthologues shared between all ruminococci genomes were mostly housekeeping genes (e.g. ribosomal proteins, tRNA synthetases) and genes involved in other highly conserved cell functions (e.g. chaperones, amino acid biosynthesis, ABC transporters). Among these orthologues, two were known carbohydrate-active enzymes (CAZymes) annotated as being involved in starch phosphorylation (Table S3): a glycogen/starch/alpha-glucan phosphorylase (*Rumal_0466*) and a maltodextrin phosphorylase (*Rumal_2782*).

Finally, we used pairwise dDDH values to estimate the number of discrete species amongst the genomes we analysed (Table S4). Of the 41 *Ruminococcus* genomes examined, we found 35 separate clades (27 in *Ruminococcaceae* and 8 in *Lachnospiraceae*) that likely represent distinct species. Among the 27 in the *Ruminococcaceae*, 4 had no current species representatives. Conversely, the *R. flavefaciens* strains separated into 13 potential species and *R. albus* separated into 5. Both *R. bromii* strains appear to delineate into two different species using this analysis.

### Expanded phylogenetic analysis describes the diversity of the ruminococci

**Fig. 2.**
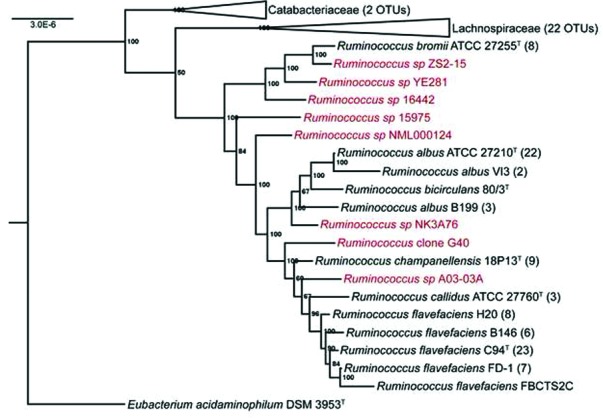
A Bayesian phylogeny of representative 16S rRNA OTUs at 97 % sequence similarity with *E. acidaminophilum* used as an outgroup. All posterior probability values are shown (ngen=10 000 000). Numbers in parentheses indicate the number of sequences that clustered into the OTU shown. Clades for *Ruminococcus* sequences that did not fall in the family *Ruminococcaceae* have been collapsed. Sequence names are coloured in black (strains of described species) and red (uncultured or undescribed strains).

With an established reference phylogeny, we sought to further probe the unexplored diversity of the genus beyond the sequenced isolates. To accomplish this, we performed a search of all 16S rRNA gene sequences available in GenBank designated as belonging to the genus *Ruminococcus*, and generated a library of 345 total sequences (Table S5), 160 of which passed our filtering criteria. Due to the nomenclature discrepancies between families in this group, this dataset contains *Ruminococcus* sequences from both the *Ruminococcaceae* and the *Lachnospiraceae*. We evaluated this sequence library using an OTU analysis, which defines phylogenetic relationships at different taxonomic levels based on the percentages of sequence identity independent of sequence counts. Our OTU analysis, as performed in mothur (Schloss *et al.*, 2009), revealed that the described species (see Fig. 1a) fell into distinct clades at 97 % 16S rRNA identity (Fig. 2). At this level, we identified 44 OTUs, 20 of which belonged to the family *Ruminococcaceae* (Table 1). Of these, eight represented potentially novel OTUs from different host sources, as well as one apparent non-host sequence from an Antarctic intertidal sediment (Yu *et al.*, 2010). As seen in our protein-based GBDP analysis (Fig. 1d), *R. albus* and *R. flavefaciens* had multiple strains that did not group into a shared OTU at a 97 % sequence similarity level.

**Table 1.** Identified 97 % similarity OTUs of GenBank *Ruminococcus* 16s rRNA gene sequences (*Ruminococcaceae* only) Representative sequence ordered by sequence source (ruminant herbivores, non-herbivores and mixed sources). Names in black represent OTUs that clustered with described *Ruminococcus* spp. strains. Names in red represent OTUs that did not cluster with described *Ruminococcus* spp. strains.

**Representative sequence name (NCBI accession no.)**	**Isolation source(s)**	**No. of sequences**
Herbivores		
*Ruminococcus flavefaciens* C94^T^ (AM915269.1)	Cow, goat, reindeer, moose, sheep	23
*Ruminococcus albus* ATCC 27210^T^ (AB538438.1)	Cow, moose, sheep, golden takin	22
*Ruminococcus flavefaciens* H20 (JF970204.1)	Cow, goat, moose	8
*Ruminococcus flavefaciens FD-1* (AM920691.1)	Cow, sheep	7
*Ruminococcus flavefaciens* B146 (AY445599.1)	Cow, addax	6
*Ruminococcus albus* B199 (AY445592.1)	Cow, sheep	3
*Ruminococcus albus* VI3 (HQ404370.1)	Sheep	2
*Ruminococcus flavefaciens* FBCTS2C (EU445111.1)	Cow	1
*Ruminococcus* sp. NK3A76 (GU324399.1)	Sheep	1
*Ruminococcus* sp. YE281 (DQ882650.1)	Cow	1
*Ruminococcus* clone G40 (JN008429.1)	Goat	1
Non-Herbivores		
*Ruminococcus champanellensis* 18 P13^T^ (AB910742.1)	Human	9
*Ruminococcus callidus* ATCC 27760^T^ (L76596.1)	Human	3
*Ruminococcus* sp. ZS2-15 (FJ889653.1)	Antarctic sandy intertidal sediment	1
*Ruminococcus bicirculans* 80/3^T^ (HF545617.1)	Human	1
*Ruminococcus* sp. 16442 (AJ318889.1)	Human	1
*Ruminococcus* sp. A03-03A (FJ542832.1)	Earthworm	1
*Ruminococcus* sp. NML000124 (EU815223.1)	Human (blood culture)	1
*Ruminococcus* sp. 15975 (AJ308104.1)	Human	1
Mixed sources		
*Ruminococcus bromii* ATCC 27255^T^ (NR025930.1)	Human, cow, pig	8

Our analysis also yielded insights into *Ruminococcus* spp. host specificity. For example, the OTU containing *R. flavefaciens* C94^T^ contained only sequences from multiple ruminants (Table 1). An identical trend was observed for the other *R. flavefaciens* strains, as well as for *R. albus* 7. Conversely, those OTUs containing *R. champanellensis*, *R. callidus* and ‘*R. bicirculans*’ contained only sequences from human sources. Finally, the OTU containing *R. bromii* ATCC 27255 showed a broader host range, including both humans, pigs and bovines.

### Environmental distribution analysis of the ruminococci

To understand the overall environmental distribution of *Ruminococcus* spp. in greater detail, we leveraged large-scale 16S rRNA-based microbiota studies from various host and non-host environments. Specifically, we aligned sequences for each study to the Silva 16S/18S NR database (SSU ref NR; release 119; 534 968 total sequences) in mothur to obtain a reference taxonomy. We examined a total of 47 studies, encompassing various types of sequencing technologies (e.g. 16S rRNA Sanger clone library, 454 pyrosequencing, Illumina) and different regions of the 16S rRNA gene (Table S6). In total, our dataset included 134 animal hosts (55 herbivores, 47 omnivores and 32 carnivores), 63 plant hosts [57 of which were from Kembel *et al.* (2014)] and 18 environmental sources (including soil, freshwater and marine environments).

**Fig. 3.**
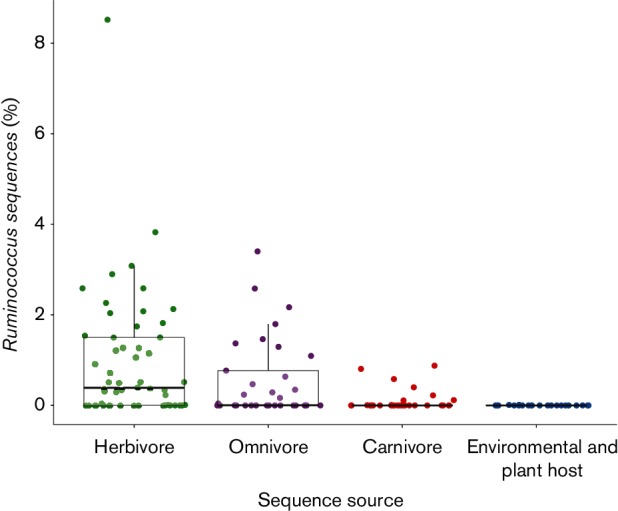
*Ruminococcus* 16S rRNA gene sequence distribution in various sequencing datasets categorized into four sample types. Each point represents the proportion of *Ruminococcus* sequences in a given dataset. Box and whisker plots are overlaid, showing the median (black bar), 75th percentile (upper hinge) and 1.5 × interquartile range (upper whisker).

From our analysis, we found that herbivores showed the highest representation of *Ruminococcus* sequences (39/55 animals examined; Fig. 3). This included expected sources such as ruminants, but also other herbivorous hosts such as avians (hoatzin), reptiles (gopher tortoise), primates (colobus monkeys, gorillas, orangutan) and other non-ruminant mammals (e.g. rhinoceros, red kangaroo, European rabbit, Linnaeus’ two-toed sloth). Omnivores also showed substantial numbers of *Ruminococcus* sequences (18/33 animals examined; Fig. 3) including humans, flying fox, ostrich and a large number of non-human primates (e.g. chimpanzee, bonobo, spider monkey and two lemur species). The carnivores also contained *Ruminococcus* sequences, although at lower percentages relative to herbivores and omnivores (11/32 animals examined; Fig. 3). One exception to this was for insectivorous/myrmecophagous mammals, some of which had sequence proportions similar to herbivores and omnivores, including the aardvark (0.42 %), the nine-banded armadillo (1.21 %) and the southern tamandua (0.61 %). Finally, we found that very few of the environmental sources contained sequences that classified to *Ruminococcus* (Fig. 3). The highest percentage of *Ruminococcus* sequences observed was 0.16 % for snow samples from a Greenland Ice Sheet (Table S7). In general, *Ruminococcus* sequence proportions were found to be significantly enriched in host-associated samples, relative to the environmental samples (Fig. 3; Fisher’s exact test, *P* value <2.2×10^−16^).

## Discussion

In this study, we used detailed single-gene-sequence-based and whole-genome-sequence-based phylogenetic analyses to assess the diversity, host specificity and environmental distribution of *Ruminococcus* spp. Having first been isolated from the bovine rumen, this genus now consists of several isolates originating from other herbivorous and omnivorous sources, leading to the hypothesis that members of this genus are strictly host associated. Although much work has been performed on the cellulolytic members of this genus to elucidate their potential roles in their given hosts, little is known about the broad distribution of this genus and the phenotypic diversity that may be present between its members.

Taken together, our phylogenetic analyses resulted in a highly resolved phylogeny that confirmed the polyphyletic nature of the genus (Fig. 1a–d). Given that several former *Ruminococcus* spp. have now been reclassified to other genera such as *Blautia* (Liu *et al.*, 2008; Lawson & Finegold, 2015), these data reinforce the need for future reclassifications of many species members (i.e. the *Ruminococcus* species within the *Lachnospiraceae*) to avoid confusion in the literature. The topology of our phylogenetic analyses also matches partial phylogenetic analyses performed for recently described species (Chassard *et al.*, 2012; Wegmann *et al.*, 2014) and offers some insights. For example, *R. bromii* appears less related to all other true ruminococci, as determined in our pairwise 16S rRNA similarity comparisons (88–89 % 16S identity; Table S8). Furthermore, it is more closely related to *Clostridium* spp., providing further evidence for the hypothesis that these organisms represent an entirely separate genus of their own (Rainey & Janssen, 1995). One possibility for this finding is that *R. bromii* may have diverged from other *Ruminococcaceae* early on, and has since become specialized. This is supported by its affinity for resistant starch substrates (Ze *et al.*, 2012) and the reduced number of CAZymes encoded by its genome, relative to other *Ruminococcus* spp., with apparent exclusivity for amylases (Ze *et al.*, 2015). Moreover, the only CAZymes shared amongst all genome sequences in our phylogenomic analysis were two genes involved in starch phosphorylation (Table S3), suggesting that the ability to utilize non-resistant dietary starches may be an ancient trait possessed by an ancestor of both *Lachnospiraceae* and *Ruminococcaceae*.

Among the other five species, two distinct clades exist: one containing *R. albus* and ‘*R. bicirculans’*, and the other containing *R. callidus*, *R. champanellensis* and *R. flavefaciens* (Fig. 1). *R. albus* and *R. flavefaciens* are distantly related (91 % 16S rRNA sequence similarity; Table S8) and coexist as important members of the rumen cellulolytic community. Previous work has shown that they possess markedly different cellulolytic strategies (Christopherson *et al.*, 2014; Dassa *et al.*, 2014) and likely compete for access to cellulosic biomass in the same niche, as *R. albus* is known to use bacteriocins to inhibit *R. flavefaciens* (Chen *et al.*, 2004). This may indicate that both species were independently acquired by ruminants, thereby reflecting their phylogenetic placement, and that the inhibitory use of bacteriocins by *R. albus* (Shi *et al.*, 1997) may serve to reinforce their distinct lineages by providing a competitive advantage within the rumen ecosystem.

In contrast, *R. callidus* and *R. champanellensis* cluster together, are more closely related (95 % 16S rRNA sequence similarity; Table S8) and are both found in the human colon. These species have different substrate preferences (Lay *et al.*, 2005; Chassard *et al.*, 2012), likely reflecting divergence and differential specialization from a common ancestor within the same host environment. One explanation for their close phylogenetic relatedness is that their divergence is more recent, and that the diversity of the human diet may have rapidly contributed to shifts in their substrate preference. Future growth experiments should be performed to assess the potential cooperation and competition between these two species.

Finally, ‘*R. bicirculans*’ appears most closely related to *R. albus* at 94 % 16S rRNA sequence similarity (Table S8). Both species inhabit different hosts(Rainey, 2009b; Wegmann *et al.*, 2014), and ‘*R. bicirculans*’ is unique from other ruminococci in that its genome consists of two chromosomes thought to be the result of a recombination event between two rRNA operons (Wegmann *et al.*, 2014). Moreover, both species prefer different but overlapping substrates, with ‘*R. bicirculans*’ selectively utilizing certain hemicelluloses but not cellulose or arabinoxylan (Wegmann *et al.*, 2014), and *R. albus* capable of utilizing a wide range of substrates, including cellulose and xylan (Christopherson *et al.*, 2014). Taken together, these observations may explain why these two species form a sister clade to the other ruminococci, as they likely diverged and became specialized within their given hosts (i.e. ruminants vs humans).

The wealth of publicly available genomes and 16S rRNA gene sequences allowed us to further explore the diversity of this genus beyond its formally described species members. Both our GBDP and OTU analyses revealed unrealized diversity at the whole-genome level as demonstrated by the dDDH values and 97 % 16S sequence similarity level, respectively. In particular, both provide evidence that many novel species of *Ruminococcus* (both cultured and uncultured) likely exist beyond those that have been described to date. For example, *R. flavefaciens* strains alone accounted for 5 OTUs at 97 % 16S rRNA sequence similarity (Fig. 2, Table 2) and 13 potential separate species using dDDH (Fig. 1d, Table S4), suggesting that *R. flavefaciens* is an extraordinarily diverse species, and that some strains may even represent separate species-level lineages. Indeed, a similar result was reported in a previous study comparing the diversity of *R. flavefaciens* strains based on *scaC* gene sequences (Jindou *et al.*, 2008). Similarly, *R. albus* accounted for three separate OTUs at 97 % sequence similarity (Fig. 2, Table 2) and five potential species using dDDH (Fig. 1d, Table S4). *R. flavefaciens* OTUs did not collapse into a single OTU until 95 % 16S rRNA sequence similarity, and *R. albus* did not do so until 92 % similarity. Furthermore, the entire genus did not fall into a single OTU until 86 % sequence similarity. Given the nature of the sequence data in our OTU analysis (i.e. a number of non-full-length 16S sequences), we chose a more conservative 97 % 16S similarity threshold for species delineation (Stackebrandt & Goebel, 1994). However, recent work suggests that even higher thresholds (e.g. 98.2–99.0 %) can be used safely depending on the particular taxonomic group (Meier-Kolthoff *et al.*, 2013b), and so we expect that the number of novel OTUs is likely to be even higher. Nevertheless, at the 97 % similarity level, the 20 ‘species-level’ OTUs in the genus *Ruminococcus* and the 27 separate species estimated using dDDH hint at the wealth of unexplored diversity that remains to be uncovered in this genus.

**Table 2.** Number of *Ruminococcus* OTUs by percentage of 16S rRNA gene sequence similarity

***Ruminococcus* OTUs (*Ruminococcaceae*)**	
16S rRNA similarity (%)	No. of OTUs
99	39
98	26
97	21
96	17
95*	11
94	9
93	8
92†	5
91	5
90‡	4
89	3
88	2
86§	1

*All *R. flavefaciens* OTUs cluster together.

†All *R. albus* OTUs cluster together.

‡All ruminococci except *R. bromii* cluster together.

§All ruminococci cluster together.

Another intriguing finding is that *R. bromii*, *R. callidus* and *R. champanellensis* fall within the *Lachnospiraceae* clade (as opposed to their usual positions in the *Ruminococcaceae*) in our GBDP nucleotide analysis (Fig. S2); however, this observation did not hold for our phylogenetic tree generated using protein-encoding genes (Fig. 1d). Although we cannot make strong conclusions due to the low branch support in the former tree, one possibility for this clading is that these particular genomes share features in common with the *Lachnospiraceae* that are not found in the *Ruminococcaceae*. This model is somewhat supported given that these particular ruminococci are all human isolates and share this feature with many of the genera within the *Lachnospiraceae* (Eren *et al.*, 2015), such as *Blautia*.

The deep sequencing of various microbiota has now become routine, and the abundance of publicly available datasets allows for the determination of *Ruminococcus* spp. distribution in various host and non-host environments. We found very few *Ruminococcus* sequences in any of the non-host datasets we examined. Indeed, no environmental dataset contained more than 0.16 % *Ruminococcus* sequences and 15 of 18 environmental datasets contained no *Ruminococcus* sequences whatsoever. The low levels of *Ruminococcus* sequences in these datasets suggests that these may belong to transient bacteria, bacteria that are not alive or may have resulted due to sequence contamination. This finding is further supported by the fact that all described ruminococci have been isolated from animal host sources, although we recognize that exhaustive culturing efforts have not been undertaken for many of the environmental samples in our dataset. Moreover, genome analysis of ruminococci like ‘*R. bicirculans*’ suggests that it has lost the ability to synthesize many essential vitamins and cofactors, while gaining other traits (e.g. bile salt hydrolases and ureases) that allow it to survive in the human gut (Wegmann *et al.*, 2014). A survey of the ruminococci in the kegg database also confirms that pathways responsible for many essential vitamin and cofactor biosynthesis are missing or incomplete in most of the genomes we examined, including those for biotin, lipoic acid, thiamine and pyridoxal phosphate (data not shown). Based on these lines of evidence, we posit that ruminococci may have evolved to survive within a host environment and have lost many of the traits required to proliferate effectively in non-host environments.

Our analysis of the deep sequence microbiota datasets also revealed that herbivores and omnivores contained the highest proportions of *Ruminococcus* sequences. Moreover, we found that the genus *Ruminococcus* has a far greater host range than has been previously reported from described isolates (Table 1). In addition to known sources like humans and ruminants, we found ruminococci in a large number of non-human primates, non-ruminant herbivores and carnivores. We also found an unexpectedly large presence of ruminococci in some insectivorous/myrmecophagous mammals. This may be due to the high abundance of chitin, the major constituent of the insect exoskeleton, which is structurally similar to cellulose. This raises the possibility that these *Ruminococcus* spp. have developed chitin degrading and fermentative abilities; thus, allowing their hosts to capitalize on this unique diet. This hypothesis is supported by the report of a *R. flavefaciens* strain with weak chitinolytic capacity (Kopecný *et al.*, 1996) and a recent metagenomic characterization of Baleen whales, which primarily eat chitin-rich crustaceans (Sanders *et al.*, 2015), that revealed an abundance of the carbohydrate-binding module family 37 enzyme that is unique to *R. albus* (Ezer *et al.*, 2008). Attempts to culture *Ruminococcus* from hosts with chitin-rich diets should be explored in the future.

In conclusion, we have presented a highly detailed view of the phylogeny, diversity and environmental distribution of the genus *Ruminococcus* using publicly available sequence datasets. In particular, we have provided evidence for the presence of potentially novel species in disparate host environments. Given the cellulolytic capability of some ruminococci, these host sources could harbour unique cellulolytic members with potential to inform advances in the production of valuable bioproducts (e.g. biofuels). Similarly, given their documented abundance in bovines and humans, a better understanding of the biology of individual species members could have important implications for animal health in general, and human health in particular. Overall, we show that this genus is extremely widespread in the animal world and more work should be undertaken to assess the roles of *Ruminococcus* spp. in their unique host environments.

## Supplementary Data

212 Supplementary File 1

## Supplementary Data

392 Supplementary File 2
