# Supplementary figures and images for: Sequence-based analysis of the genus Ruminococcus resolves its phylogeny and reveals strong host association
Source: Microb Genom. 2016 Dec 12;2(12):e000099. doi: 10.1099/mgen.0.000099 (PMC5359413; doi:10.1099/mgen.0.000099)

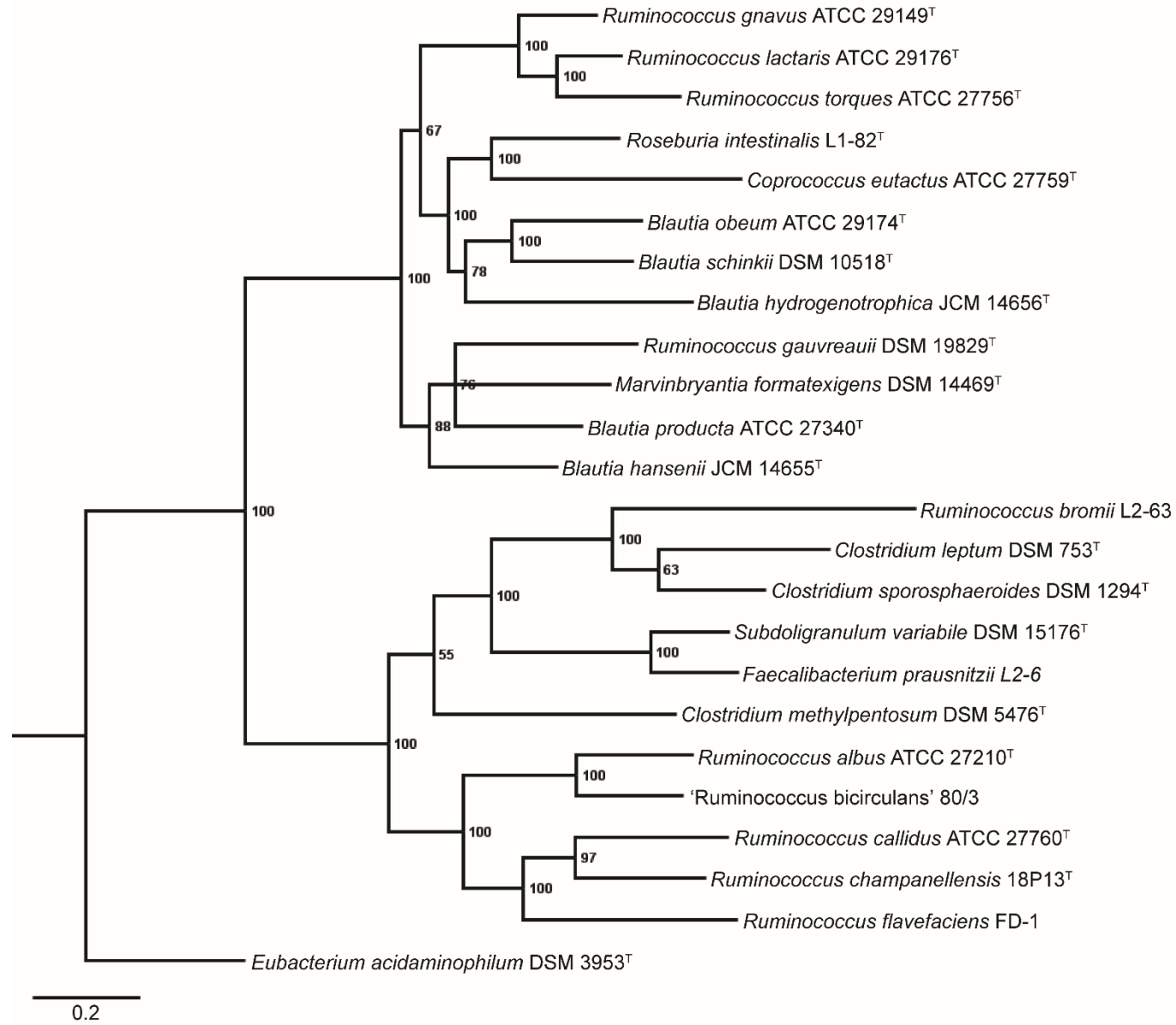

Lachnospiraceae

Ruminococcaceae

■ \*

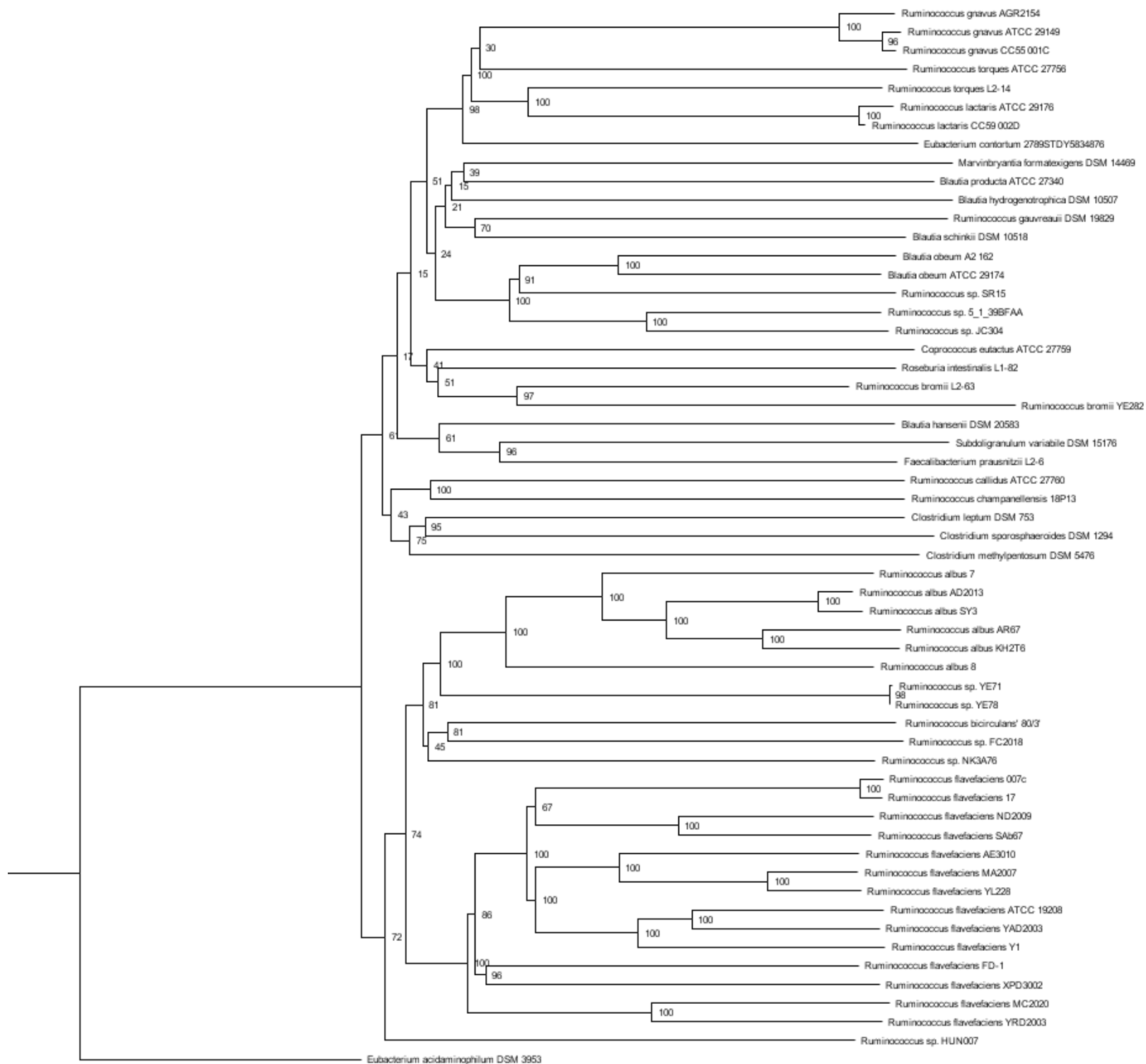

Supplement: Supplementary File 2 [file mgen-02-99-s002.pdf]
